# Impact of different consensus definition criteria on sepsis diagnosis in a cohort of critically ill patients—Insights from a new mathematical probabilistic approach to mortality-based validation of sepsis criteria

**DOI:** 10.1371/journal.pone.0238548

**Published:** 2020-09-08

**Authors:** Franz-Simon Centner, Jochen J. Schoettler, Anna-Meagan Fairley, Holger A. Lindner, Verena Schneider-Lindner, Christel Weiss, Manfred Thiel, Michael Hagmann

**Affiliations:** 1 Department of Anaesthesiology and Surgical Intensive Care Medicine, University Medical Center Mannheim, Medical Faculty Mannheim of Heidelberg University, Mannheim, Germany; 2 Department of Community Health Sciences, University of Manitoba, Winnipeg, Canada; 3 Department of Biomedical Informatics of the Heinrich-Lanz-Center, Medical Faculty Mannheim of Heidelberg University, Mannheim, Germany; 4 Interdisciplinary Center for Scientific Computing, Heidelberg University, Heidelberg, Germany; Wayne State University, UNITED STATES

## Abstract

**Background:**

Sepsis-3 definition uses SOFA score to discriminate sepsis from uncomplicated infection, replacing SIRS criteria that were criticized for being inaccurate. Eligibility of sepsis-3 criteria for sepsis diagnosis and the applied validation methodology using mortality as endpoint are topic of ongoing debate. We assessed the impact of different criteria on sepsis diagnosis in our ICU and devised a mathematical approach for mortality-based validation of sepsis criteria. As infectious status is often unclear at clinical deterioration, we integrated non-infected patients into analysis.

**Methods:**

Suspected infection, SOFA and SIRS were captured for an ICU cohort of a university center over one year. For raw scores (SIRS/SOFA) and sepsis criteria (SIRS≥2/SOFA≥2/SOFA_change≥2) frequencies and associations with in-hospital mortality were assessed. Using a mathematical approach, we estimated the correlation between sepsis and in-hospital mortality serving as reference for evaluation of observed mortality correlations of sepsis criteria.

**Results:**

Of 791 patients, 369 (47%) were infected and 422 (53%) non-infected, with an in-hospital mortality of 39% and 15%. SIRS≥2 indicated sepsis in 90% of infected patients, SOFA≥2 in 99% and SOFA_change≥2 in 77%. In non-infected patients, SIRS, SOFA and SOFA_change were ≥2 in 78%, 88% and 58%. In AUROC analyses neither SOFA nor SIRS displayed superior mortality discrimination in infected compared to non-infected patients. The mathematically estimated correlation of sepsis and in-hospital mortality was 0.10 in infected and 0 in non-infected patients. Among sepsis criteria, solely SIRS≥2 agreed with expected correlations in both subgroups (infected: r = 0.19; non-infected: r = 0.02).

**Conclusions:**

SOFA≥2 yielded a more liberal sepsis diagnosis than SIRS≥2. None of the criteria showed an infection specific occurrence that would be essential for reliable sepsis detection. However, SIRS≥2 matched the mortality association pattern of a valid sepsis criterion, whereas SOFA-based criteria did not. With this study, we establish a mathematical approach to mortality-based evaluation of sepsis criteria.

## Introduction

Sepsis is a dysregulated systemic inflammatory and immune response to microbial invasion [1] and the primary cause of death from infection [2]. As mortality increases with delay in treatment [3–5] the diagnosis of sepsis mandates immediate administration of broad spectrum antibiotics according to Surviving Sepsis Campaign guidelines [6]. On the other hand, the non-indicated use of broad spectrum antibiotics has serious adverse consequences for both the population and the individual patient, as it is connected to the emergence of multi-drug resistant bacteria and morbidity caused by side effects [7, 8]. Therefore an accurate sepsis diagnosis is of crucial importance [2].

As a gold standard diagnostic test for sepsis is still missing, clinical sepsis criteria serve to diagnose sepsis and differentiate it from ‘uncomplicated infection’ [2, 9]. Because the formerly used Systemic Inflammatory Response Syndrome (SIRS) criteria (sepsis-1 and -2) [10, 11] were criticized for occurring too frequently among infected and non-infected patients and their eligibility as sepsis criteria was questioned [2, 12, 13], new clinical sepsis criteria were introduced by a consensus definition of the Society of Critical Care Medicine (SCCM) and the European Society of Intensive Care Medicine (ESICM) in 2016, referred to as sepsis-3. Accordingly, a patient with a (suspected) infection and an acute change in Sequential Organ Failure Assessment (SOFA) score of ≥2 points is diagnosed as septic [2]. A large validation study which assessed the predictive power for in-hospital mortality as primary outcome showed stronger discrimination for in-hospital mortality of SOFA and an acute change in SOFA compared to SIRS in infected patients [9]. Sepsis-3 authors argued that this proved superior validity of SOFA-based criteria for sepsis diagnosis [2, 9]. Although these new criteria were introduced in order to provide better diagnostic guidance for sepsis, in the original sepsis-3 ICU cohort, application of SOFA≥2 resulted in a higher sepsis frequency than SIRS≥2 (91% versus 84% [9]), which is in conflict with the intention to generate a more specific criterion.

In clinical practice not only the differentiation between ‘uncomplicated infection’ and sepsis is challenging. Additionally, by the time a patient develops signs of organ dysfunction or systemic inflammation it is often unclear whether these conditions are related to an infection or not [2, 14–16]. Ideal sepsis criteria should therefore not only differentiate between patients with sepsis and with uncomplicated infection, but also help to separate patients who are critically ill due to sepsis from those suffering from non-infectious conditions. The ability of sepsis-3 criteria to make this differentiation was not analyzed in the sepsis-3 validation study, which limited analysis to the subgroup of infected patients. We integrated non-infected patients into analysis, thereby addressing this named limitation of sepsis-3 [2, 9].

In this context, we designed this study to contribute to the evaluation of sepsis criteria in the ICU setting by addressing the following issues:

- We assessed the impact of the application of sepsis-3 versus sepsis-1/2 clinical criteria in an ICU cohort, especially on the frequencies of sepsis and the differentiation between uncomplicated infection and sepsis.
- As the use of mortality as endpoint for validation of sepsis criteria in sepsis-3 [2, 9] has been criticized [17–21] we developed a mathematical approach to reasonably use mortality for the validity evaluation of sepsis criteria. Thereby, we estimated the correlation between sepsis and in-hospital mortality, which enabled the introduction of quantitative references for mortality-based evaluation of sepsis criteria.
- By including non-infected patients in our study, we were able to analyze whether the occurrence or the mortality association of the proposed clinical sepsis criteria differ dependent on the infectious status of a patient. Applying our probabilistic approach, we investigated if the strength of the mortality association of the proposed clinical sepsis criteria was within the mathematically estimated range for infected and non-infected patients.

## Materials and methods

### Study design and settings

This prospective observational study [22] was conducted at the 25-bed ICU of the Department of Anaesthesiology and Surgical Intensive Care Medicine at University Medical Center Mannheim. All encounters (age ≥18 years) with complete ICU stay between June 1st 2016 and July 9th 2017 were included. Starting date was set due to introduction of daily SOFA scoring in reaction to sepsis-3 [2, 9]. The Ethics Commission II of Medical Faculty Mannheim approved the study (2016-800R-MA) and waived the need for informed consent.

### Measurements and definitions

SOFA scores [23] were determined daily by intensivists, and SIRS criteria [10] were extracted by computational query from the electronic health record. For identification of suspected infection and its onset the approach chosen in sepsis-3 validation study was applied [9] and sepsis-3 criteria were evaluated in the same time frames around infection onset [9] (For further details on all measures, see the S1 Appendix section A: Details on Definitions). To define equivalent evaluation time points for non-infected patients, these were selected by computational query to achieve a distribution similar to infection onset in infected patients (Fig A in S1 Appendix section A). We applied maximum SOFA and acute change in SOFA (henceforth referred to as ‘SOFA’ and ‘SOFA_change’) as closely as possible to sepsis-3 implementation [9] (For details see S1 Appendix section A). As it was considered closest to the sepsis-1/2 definition [10, 11] the 24 hour window before infection onset was chosen for SIRS analyses.

### Statistical analysis

Statistical analysis was operated with R 3.3.2 [24] and the pROC package [25]. A p-value ≤0.05 was regarded statistically significant. No adjustment for multiplicity was applied.

#### Assessment of the impact of different sepsis criteria on sepsis diagnosis

Raw scores (SIRS/SOFA) and the proposed sepsis criteria (SIRS≥2/SOFA≥2/SOFA_change≥2) were analyzed separately for infected and non-infected patients. For scores and in-hospital mortality, Area Under the Receiver Operating Characteristic Curve (AUROC) analyses was performed, applying similar baseline risk modeling as in sepsis-3 [9]. We calculated the discrimination capacity of score plus baseline risk model and score alone (For details see S1 Appendix section B). The latter was chosen for primary reporting because the baseline model variables are not part of sepsis-3 criteria. The score threshold of 2 was evaluated by sensitivity, specificity, positive and negative predictive value, risk ratio and odds ratio for in-hospital mortality.

#### Investigating the correlation between sepsis and (in-hospital) mortality

In a ‘framework for the development and interpretation of different sepsis definitions and clinical criteria’, sepsis-3 authors defined criterion validity as the extent to which a proposed measure (clinical criteria) of a disease (sepsis) agrees with an existing accepted measure (in-hospital mortality) [26, 27]. They highlighted the importance to set expectations about this agreement when assessing validity. Nevertheless, it was omitted during the sepsis-3 validation process to formulate an expectation for the agreement between sepsis and in-hospital mortality [9]. Instead, it was assumed to be rather large [9]. We replaced this assumption with an empirical estimate using sepsis-3 validation study data [9] in conjunction with SOFA ≥2 as septic condition to determine the septic status of a patient. This approach yielded an expected correlation between sepsis and in-hospital mortality of 0.10 for infected patients (Details on the probabilistic mathematical model of the relation between sepsis and in-hospital death and the performed calculations can be found in the S1 Appendix section D and S2 Appendix). This value served as reference for statistical examination of the observed correlations in our cohort. As sepsis cannot cause death in non-infected patients, the correlation between in-hospital mortality and sepsis must be 0 in this subgroup. This facilitated the investigation of an infection-specific mortality association pattern that must be displayed by valid sepsis criteria. Pearson correlation coefficients were calculated to assess mortality associations and statistical examination of correlations was based on refined Fisher transformation [28].

#### Sensitivity analysis

For sensitivity analyses, examination was limited to the day of ICU admission for two reasons: It entailed timely fixation for comparison between infected and non-infected patients, and major studies examining sepsis-3 in the ICU limited observation to the 24 hours after admission [29–33] (for details see S1 Appendix section C).

## Results

### Frequencies of proposed sepsis criteria in our ICU cohort and descriptive results in relation to in-hospital mortality

**Fig 1:**
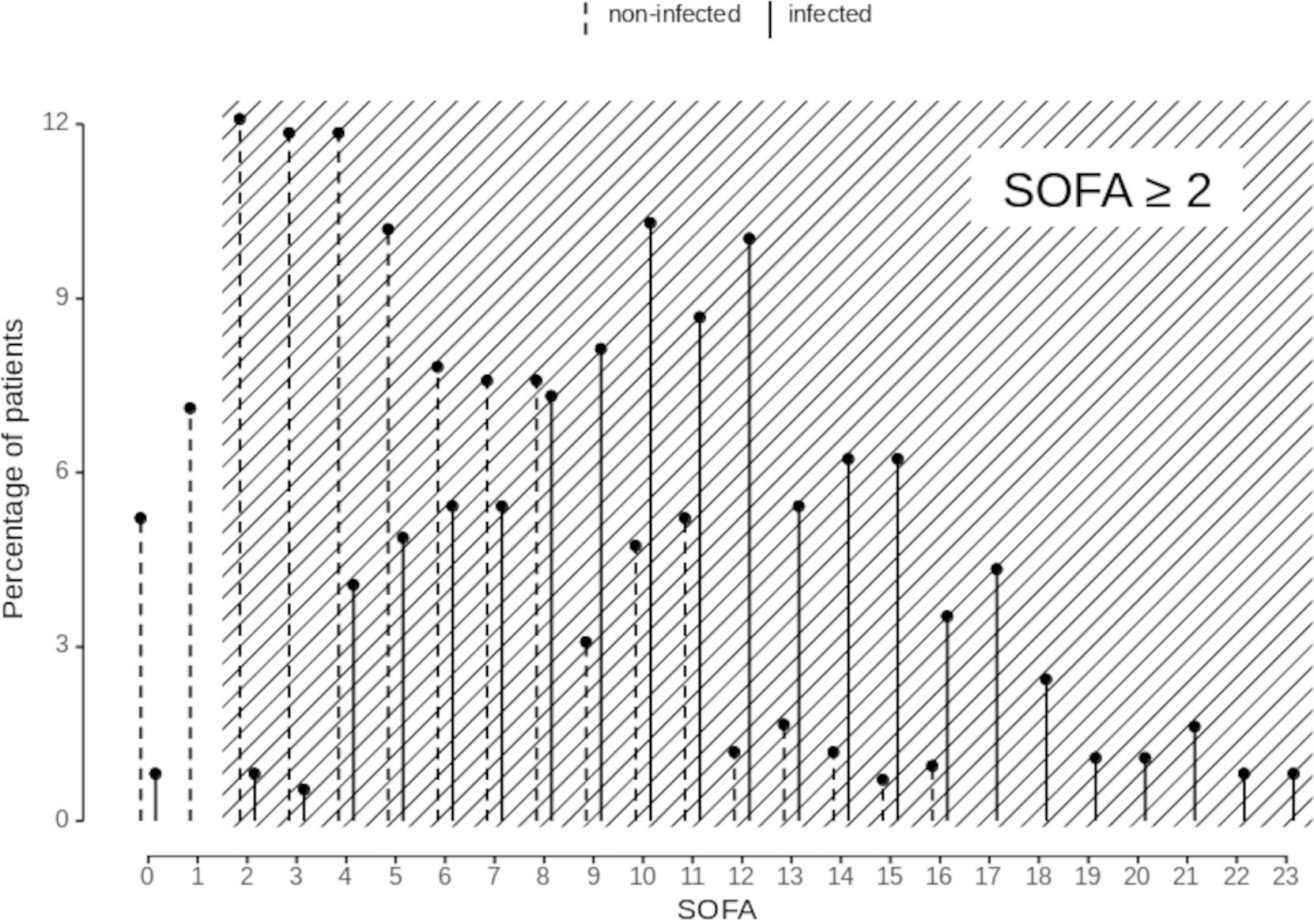
Distribution of maximum Sequential Organ Failure Assessment (SOFA) scores among infected patients (n = 369) at infection onset and non-infected patients (n = 422) at evaluation time points. All scores on shaded background fulfill the threshold ≥2 proposed by sepsis-3 as clinical sepsis criterion. SOFA was ≥2 in 93% of all patients (infected: 99%; non-infected patients: 88%). The x-axis is the score range, with SOFA truncated at 23 points for illustration.

Of 791 examined patients, 369 (47%) were assigned infected and 422 (53%) non-infected, with an in-hospital mortality of 39% and 15% respectively (Table 1). SOFA distribution at infection onset and evaluation time points is displayed in Fig 1 (for definition of infection onset and evaluation time points see S1 Appendix section A). Frequencies of positive sepsis criteria by reference population are shown in Table 2. Frequencies of sepsis diagnoses in dependence on applied sepsis criteria in the overall ICU cohort were 42% (SIRS≥2), 46% (SOFA≥2), and 36% (SOFA_change≥2). In infected patients, SIRS≥2 indicated sepsis in 90% at infection onset, SOFA≥2 in 99% and SOFA_change≥2 in 77%. In non-infected patients at the respective evaluation time points, SIRS, SOFA and SOFA_change were ≥2 in 78%, 88% and 58% respectively. Further, SIRS was ≥2 in 5231 of 5978 (88%) observed patient days in the infected group, and in 1292 of 1724 days (75%) in the non-infected group. The respective percentages for SOFA≥2 were 100% in infected and 94% non-infected patients respectively (for SOFA_change 12% and 23%, respectively) (Table 3).

**Table 1:** Characteristics of the Intensive Care Unit (ICU) cohort by infectious status (n = 791).

Descriptive variable	Infected	Non-infected
Number of encounters, n (%)	369 (47)	422 (53)
Age, mean (sd), years	63 (15)	62 (16)
Male, n (%)	243 (66)	225 (53)
SOFA, mean (sd)	11.0 (4.5)^a^	5.4 (3.6) ^b^
SIRS^a^, mean (sd)	2.7 (0.9) ^a^	2.2 (1.0) ^b^
Weighted Charlson comorbidity index, median (Q1 –Q3)	3 (2–4)	3 (2–4)
ICU length of stay, mean (sd), days	16.2 (15.7)	4.1 (4.5)
In-hospital mortality, n (%)	144 (39)	63 (15)

ICU, Intensive Care Unit; Q1, 25%-percentile; Q3, 75%-percentile; sd, standard deviation; SIRS, Systemic Inflammatory Response Syndrome; SOFA, Sequential Organ Failure Assessment.

^a^ at infection onset.

^b^ at defined evaluation time points for non-infected patients (see Fig A in S1 Appendix section A).

**Table 2:** Frequencies of positive sepsis criteria (SIRS≥2/SOFA≥2/SOFA_change≥2) depending on reference population.

	SIRS≥2	SOFA≥2	SOFA_change≥2
overall ICU cohort n = septic^a^/N = overall, (%)	333/791 (42)	366/791 (46)	285/791 (36)
infected patients n = septic^a^/N = infected, (%)	333/369 (90)	366/369 (99)	285/369 (77)
non-infected patients n = criteria positive^b^/N = non-infected, (%)	329/422 (78)	370/422 (88)	245/422 (58)

ICU, Intensive Care Unit; SIRS, Systemic Inflammatory Response Syndrome; SOFA, Sequential Organ Failure Assessment.

^a^ at infection onset.

^b^ at defined evaluation time points for non-infected patients (see Fig A in S1 Appendix section A).

**Table 3:** Frequencies of positive sepsis criteria (SIRS≥2/SOFA≥2/SOFA_change≥2) per observed patient day depending on reference population.

	SIRS≥2	SOFA≥2	SOFA_change≥2
infected patients n = septic days/N = patient days of infected patients, (%)	5231/5978 (88)	5972/5978 (100)	739/5978 (12)
non-infected patients n = criteria positive days/N = patient days of non-infected patients, (%)	1292/1724 (75)	1627/1724 (94)	392/1724 (23)

ICU, Intensive Care Unit; SIRS, Systemic Inflammatory Response Syndrome; SOFA, Sequential Organ Failure Assessment.

Regarding the relation of proposed sepsis criteria to in-hospital mortality (Table 4), at infection onset, SIRS displayed a sensitivity of 97% [95% Confidence Interval (CI) 94%–99%] and specificity of 14% [10%–19%] for in-hospital mortality. SOFA was ≥2 in 100% of decedents and in 99% of survivors, resulting in a sensitivity of 100% [100%–100%] and specificity of 1% [0%–3%] for in-hospital mortality at infection onset. In non-infected patients the respective values were for SIRS≥2 79% [68%-89%] and 22% [18%-27%] and for SOFA≥2 100% [100%-100%] and 14% [11%-18%] respectively. Positive and negative predictive values for in-hospital mortality in infected patients for SIRS≥2 were 42% and 89%, and for SOFA≥2 39% and 100% respectively. In non-infected patients positive and negative predictive values for SIRS≥2 were 15% and 86% and for SOFA≥2 17% and 100% respectively. Risk ratio and odds ratio for in-hospital mortality were significant for SIRS≥2 in infected patients at infection onset (risk ratio: 3.8 [1.4–9.7]; odds ratio: 5.8 [2.0–16.8]), but not significant in non-infected patients (risk ratio: 1.1 [0.6–2.0]; odds ratio: 1.1 [0.5–2.2]). Because all deceased patients had a SOFA score ≥2 at time points of infection or evaluation, risk and odds ratios could not be calculated for SOFA≥2. Risk ratio for in-hospital mortality of SOFA_change≥2 was similar in infected and non-infected patients (2.2 [1.4–3.5]) and 2.2 [1.2–3.6] respectively). Of note, 20% of infected patients that had a change in SOFA <2 points at infection onset died in hospital later on. In-hospital-death was observed for 11% of infected patients with SIRS <2 at infection onset.

**Table 4:** Descriptive results applying a cutoff ≥2 for SIRS, SOFA and SOFA_change in the study cohort by infectious status and in relation to in-hospital mortality.

		Infected (n = 369)			Non-infected (n = 422)		
		SIRS	SOFA	SOFA_change	SIRS	SOFA	SOFA_change
Sensitivity^a^, % (95%CI)		97 (94–99)	100 (100–100)	88 (83–93)	79 (68–89)	100 (100–100)	75 (63–86)
Specificity^a^, % (95%CI)		14 (10–19)	1 (0–3)	30 (24–36)	22 (18–27)	14 (11–18)	45 (40–50)
Positive Predictive Value^a^, % (95%CI)		42 (41–44)	39 (39–40)	45 (42–47)	15 (13–17)	17 (16–18)	19 (17–22)
Negative Predictive Value^a^, % (95%CI)		89 (77–98)	100(100–100)	80 (72–87)	86 (80–92)	100 (1 00–100)	91 (87–95)
Deceased in Hospital, n (%)	≥2	140 (42)	144 (39)	127 (45)	50 (15)	63 (17)	47 (19)
	<2^b^	4 (11)	0 (0)	17 (20)	13 (14)	0 (0)	16 (9)
Risk Ratio^a^ (95%CI)		3.8 (1.4–9.7)	-	2.2 (1.4–3.5)	1.1 (0. 6–2.0)	-	2.2 (1.2–3.6)
Odds Ratio^a^ (95%CI)		5.8 (2.0–16.8)	-	3.2 (1.7–5.7)	1.1 (0.5–2.2)	-	2.4 (1.3–4.4)

Results are reported for time point of infection onset or evaluation time points in non-infected patients and a cutoff ≥2. CI, Confidence Interval; ICU, Intensive Care Unit; SIRS, Systemic Inflammatory Response Syndrome; SOFA, Sequential Organ Failure Assessment.

^a^ for in-hospital mortality.

^b^ Denominator is the number of patients with cutoff <2 (for infected patients: SIRS: n = 36; SOFA: n = 3; SOFA_change: n = 84; for non-infected patients: SIRS: n = 93; SOFA: n = 52; SOFA_change: n = 177).

### Discrimination of raw scores (SIRS/SOFA) for in-hospital mortality

**Fig 2:**
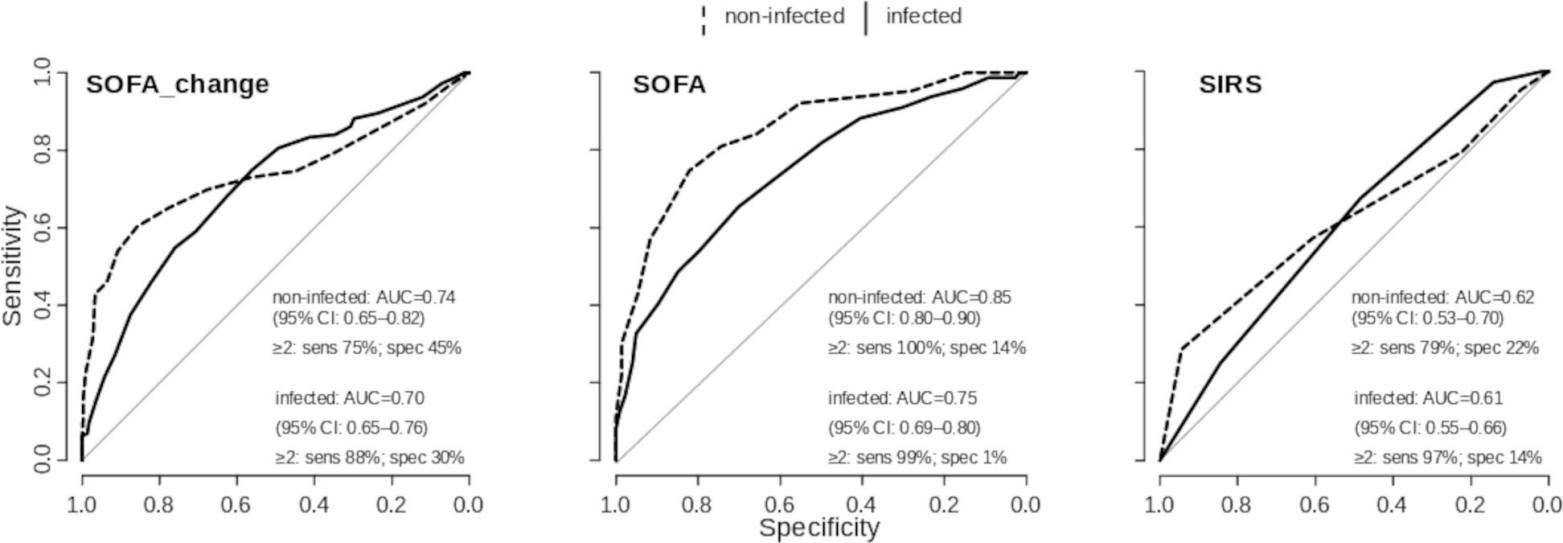
Receiver Operating Characteristic (ROC) curves for Sequential Organ Failure Assessment (SOFA) score applications and Systemic Inflammatory Response Syndrome (SIRS) criteria discrimination for in-hospital mortality in infected (n = 369) and non-infected (n = 422) patients. Discrimination capacity of SOFA for in-hospital mortality was significantly stronger in non-infected than in infected patients (p = 0.006). SIRS discriminated for in-hospital mortality in both subgroups with lower AUC compared to SOFA applications. If applied with the proposed threshold, SOFA≥2 was more sensitive and less specific for in-hospital mortality than SIRS≥2. AUC: Area Under the Curve; SIRS: Systemic Inflammatory Response Syndrome; SOFA: Sequential Organ Failure Assessment; sens: sensitivity; spec: specificity.

AUROC of SOFA for in-hospital mortality was 0.75 ([95%CI 0.69–0.80]; p<0.001) for infected patients, and significantly higher for non-infected patients 0.85 ([0.80–0.90]; p<0.001) (Fig 2). SOFA_change discriminated for in-hospital mortality similarly in infected (AUROC 0.70 [0.65–0.76]; p<0.001) and non-infected patients (AUROC 0.74 [0.65–0.82]; p<0.001). SIRS was associated with in-hospital mortality in both subgroups with lower AUROCs compared to SOFA and SOFA_change (AUROC infected: 0.61 [0.55–0.66]; p<0.001; non-infected: 0.62 [0.53–0.70]; p = 0.002) (Fig 2).

### Correlation of raw scores (SIRS/SOFA) and sepsis criteria (SIRS≥2/SOFA≥2/SOFA_change≥2) with in-hospital mortality in relation to expected correlations between sepsis and in-hospital mortality

The expected correlation between sepsis and in-hospital mortality was 0.10 for infected and 0 for non-infected patients (details on the probabilistic mathematical model can be found in S1 Appendix section D). As a raw score, without a threshold, the observed correlation of SOFA with in-hospital mortality was significantly stronger than the expected correlation for a sepsis criterion in both subgroups (infected: r = 0.43, p<0.01; non-infected: r = 0.50, p<0.01). Correlation of SIRS and in-hospital mortality was scarcely in range of expectations in infected patients (r = 0.20; p = 0.0504) (Table 5).

**Table 5:** Observed correlations of raw scores (SIRS/SOFA) and sepsis criteria (SIRS≥2/SOFA≥2/SOFA_change≥2) with in-hospital mortality in comparison to the estimated expected correlation of sepsis and in-hospital mortality.

	Expected correlation^a^	Observed correlations with mortality				
	Sepsis—mortality	SIRS	SOFA	SIRS≥2	SOFA≥2	SOFA_ change≥2
Non-infected	0	0.16 (p<0.01)^b^	0.50 (p<0.01)^b^	**0.01 (p = 0.77)**^**c**^	0.16 (p<0.01)^b^	0.14 (p<0.01)^b^
Infected	0.10	**0.20 (p = 0.0504)**^**c**^	0.43 (p<0.01)^b^	**0.19 (p = 0.10)**^**c**^	**0.07 (p = 0.54)**^**c**^	0.21 (p = 0.04)^b^

SIRS, Systemic Inflammatory Response Syndrome; SOFA, Sequential Organ Failure Assessment.

^a^ Based on mathematical reasoning underlying sepsis-3 validation study numbers for infected ICU patients (eTable 3 in supplement of [9]; SOFA≥2 was taken as septic condition. For calculation details see S2 Appendix). Details on the probabilistic model of the relation between sepsis and in-hospital death can be found in S1 Appendix section D.

^b^ If p was ≤0.05 the correlation of the sepsis criterion with in-hospital mortality differed significantly from the expected correlation between sepsis and mortality.

^c^ If p was >0.05 the correlation was in range of the expected correlation highlighted in bold.

Examining the threshold of 2, SIRS≥2 agreed with expected correlations in both subgroups (infected: r = 0.19, p = 0.10; non-infected: r = 0.01, p = 0.77). SOFA≥2 was in range of expectations in infected patients, but deviated significantly from the expected mortality association pattern in non-infected patients (r = 0.16, p<0.01). SOFA_change≥2 deviated from expectations in both patient subgroups (infected: r = 0.21, p = 0.04; non-infected: r = 0.14, p<0.01) (Table 5).

### Sensitivity analysis

Sensitivity analysis restricting examination to ICU admission showed that 261 patients (33% of total cohort) entered the ICU with an infection (in-hospital mortality 44%). Results were overall consistent with those reported for the entire cohort (details can be found in S1 Appendix section C).

## Discussion

In this study, we assessed the impact of sepsis-3 criteria on sepsis diagnosis in comparison to sepsis-1/2 criteria within an ICU cohort and we performed a new, reference-guided mathematical approach to mortality-based validation of sepsis criteria.

Major findings of our study were: i) Compared to SIRS≥2, SOFA≥2 was more frequently observed in infected and non-infected patients at infection onset or the corresponding evaluation time points. The same was true for respective comparisons based on patient days (Tables 2 and 3). ii) Only SIRS≥2 showed significantly high risk ratio and odds ratio for in-hospital mortality in infected but not in non-infected patients, whereas risk ratio of SOFA_change≥2 was similar in both patient subgroups and SOFA ≥2 was met by all non-survivors in both patient groups preventing mortality risk prediction even in non-infected patients (Table 4). iii) In-hospital mortality association evaluated by AUROC analyses showed higher values for SOFA applications compared to SIRS score but neither displayed higher values for infected than for non-infected patients. In contrast, AUROC of SOFA was even significantly higher in non-infected compared to infected patients (Fig 2). And finally iv) Solely SIRS≥2 but neither SOFA≥2 nor SOFA_change≥2 met the pattern and strength of correlation with in-hospital mortality that would be expected of a valid sepsis criterion (Table 5) as estimated by our probabilistic mathematical model (see S1 Appendix section D) developed in response to the sepsis-3 criterion validity evaluation approach, as previously requested [9, 26, 27].

Based on these findings we advise caution to consider SOFA-based sepsis criteria to be of superior validity as compared to SIRS-based sepsis criteria in diagnosing sepsis in critically ill patients regarding the additional analytical aspects found in this study and discussed in the following:

### SOFA application: Maximum versus change

Main analyses in the sepsis-3 validation study were undertaken and reported for SOFA≥2 [9], while investigation of an acute change in SOFA ≥2 resulted from a post hoc analysis [9]. Nevertheless an acute change in SOFA ≥2 was chosen as final criterion [2]. Because a baseline SOFA score to calculate a change from can be unknown in clinical practice and research datasets, especially around hospital and ICU admission respectively, the task force suggested to assume the baseline SOFA score to be zero in patients not known to have preexisting organ dysfunction [2]. In the original sepsis-3 ICU cohort, onset of infection happened within 48 hours of admission in 77% [9]. Dependent on how often 0 was assumed as baseline SOFA score, which was not reported, SOFA≥2 and SOFA_change≥2 lead to more or less similar results. Later it was stated by the corresponding author of the sepsis-3 definitions that two SOFA points were sufficient to meet criteria for sepsis, and that there was no requirement to calculate a change [2, 34]. This results in heterogeneous SOFA operationalizations in studies using sepsis-3 clinical criteria for sepsis diagnosis in the ICU: Major studies primarily analyzed SOFA≥2 [9, 32, 33]. In other studies the used sepsis-3 implementation is called “an acute change in SOFA≥2” but zero is used as baseline SOFA without taking preexisting organ dysfunction into account, consequently the used criterion is equivalent to SOFA≥2 [29, 30, 35]. Others found individual solutions to operationalize preexistent organ dysfunction for calculation of “an acute change in SOFA≥2” as primary analyzed criterion [31, 36] or in sensitivity analyses [29, 30, 32]. In some studies, it is not documented how the “acute change in SOFA” was implemented [21, 37]. This heterogeneity in operationalization of sepsis-3 criteria is problematic, as variation in sepsis defining criteria and their application have been identified as major obstacle in sepsis reporting [38, 39]. In our study we analyzed both, SOFA≥2 and SOFA_change≥2, which revealed that, depending on the performed analysis, the two forms of SOFA application captured discrepant results and thus cannot be used interchangeably. While discrimination for in-hospital mortality of SOFA and SOFA_change were similar in the sepsis-3 validation study and our ICU cohort (AUROC for SOFA and SOFA_change respectively: 0.75 and 0.70 in our data while 0.74 and 0.70 in sepsis-3 [9]), consecutive frequencies of sepsis diagnosis differed significantly in our study: SOFA_change≥2 assigned 22% less infected patients as septic compared to SOFA≥2 (Table 2).

For reasons of comparability, in the following section we focus the discussion on SOFA≥2, because main results of the sepsis-3 validation study concerning the ICU are reported for this SOFA application [9] and the majority of studies analyzing sepsis-3 criteria in the ICU in fact studied SOFA≥2 [9, 29, 30, 32, 33, 35].

### Impact of SOFA≥2 as criterion in infected patients

In our cohort, SOFA was ≥2 in 99% of infected patients at infection onset, SIRS in 90% (Table 2). Consequently, SIRS≥2 indicated discrimination between uncomplicated infection and sepsis in 10 of 100 infected ICU patients in our study, SOFA≥2 only in 1 of 100. Hence, SOFA≥2 diminished this differentiation, which significantly impacts epidemiological figures and potential clinical consequences: Also in the original sepsis-3 ICU cohort, SOFA≥2 resulted in a higher frequency of sepsis diagnoses than SIRS≥2 at infection onset (91% and 84%) [9], which was not discussed. Likewise, for mixed and cancer patients admitted to ICUs with infection, higher occurrence of SOFA≥2 (90%, 97% and 87%) compared to SIRS≥2 (87%, 77% and 59%) was reported [29, 31, 35]. SOFA≥2 resulted in an increase in sepsis diagnoses of 4% compared to SIRS≥2 in our overall cohort, and of 9% within the subgroup of infected patients (Table 2). Fittingly, Fullerton et al. reported an increase in sepsis incidence of 4% and 28% respectively regarding comparable ICU populations [35]. These higher frequencies of sepsis diagnoses were observed although sepsis-1/2 criteria for ‘simple’ sepsis [10, 11], not ‘severe sepsis’, were applied. Considering that sepsis-3 sought to require the presence of organ dysfunction and was thought to replace the former ‘severe sepsis’ [2], the increase in sepsis diagnoses according to SOFA≥2 contradicts expectations [18, 19, 40–43], reinforcing the question whether SOFA along with the threshold of 2 was an adequate operationalization of organ dysfunction [21, 31, 44–46]. This is of special interest, as the importance of considering patients’ severity of illness when deciding about the breadth of antibiotics has been highlighted [47, 48] and studies document that patients with less severe disease suffer from negative consequences of antibiotic overuse [8, 47, 49, 50]. Our investigation raises concerns in that a switch from SIRS≥2 to SOFA≥2 as underlying criterion may increase the frequency of sepsis diagnoses in the ICU.

### Infection-specific occurrence of sepsis criteria

By the time a patient develops signs of an organ dysfunction or systemic inflammation it is often unclear whether these conditions are related to an infection or not [2, 14–16]. An infection-specific occurrence would therefore be essential for a reliable sepsis criterion [51]. In our study, none of the evaluated criteria displayed this desirable pattern of occurrence. SOFA was ≥2 on 100% (SIRS: 88%) of observed days in infected patients and on 94% (SIRS: 78%) in non-infected patients (Table 3). A major argument to replace SIRS as sepsis criterion had been that it was present in too many patients, including those who never develop infection [2, 12]. Therefore, Singer et al. denied SIRS’ validity as sepsis criterion. In our cohort, these concerns are even more substantial for SOFA≥2. Fittingly, before sepsis-3 it was evident that SOFA, as raw score, captures adverse outcome [14, 52–54] and organ dysfunction [14, 23], but irrespective of the cause [14, 42, 55] and in particular irrespective of the presence of sepsis [14]. This gave reason to rename SOFA from ‘Sepsis-related Organ Failure Assessment’ to ‘Sequential Organ Failure Assessment’ [14]. If a patient is severely ill, may this be evident because of signs of organ dysfunction or systemic inflammation or both, one of the crucial clinical question remains whether this condition is caused by an infection or not [16, 56]. Our study underlines that neither of the proposed sepsis criteria are helpful to answer this question (compare frequencies of SIRS and SOFA based sepsis criteria in infected and non-infected patients (Tables 2 and 3)), as also others have highlighted [12, 14, 51].

### Associations of sepsis criteria with in-hospital mortality

It was the pivotal effort of the sepsis-3 validation strategy to demonstrate superior criterion validity of SOFA as sepsis measure by showing a stronger association with in-hospital mortality for SOFA than SIRS in infected patients, measured by AUROC [2, 9]. In very good agreement with sepsis-3 validation study, we observed moderate discrimination capacity of SOFA applications for in-hospital mortality in infected patients (AUROC for SOFA and SOFA_change respectively: 0.75 and 0.70 in our data while 0.74 and 0.70 in sepsis-3 [9]) that was higher than SIRS’ AUROC for in-hospital mortality. Further studies in the ICU setting replicating this evaluation approach also reported comparable results [29, 31, 32, 37]. However, the use of AUROC for the assessment of risk prediction in general [57] and for sepsis-3 validation in particular [17, 58] was criticized. AUROC is a measure for discrimination, but it can lead to false conclusions if it is used to assess predictive correctness [57]. Moreover, AUROC analyses of neither SOFA nor SIRS displayed superior discrimination for in-hospital mortality in infected compared to non-infected patients. AUROC of SOFA for in-hospital mortality was even significantly higher in non-infected patients than in infected patients (Fig 2). Thus, neither SOFA nor SIRS, as raw scores, help to discriminate infectious from non-infectious causes of mortality in critically ill patients. Notably, AUROC assesses SOFA and SIRS as raw scores and carries no information about the proposed sepsis criteria threshold for SIRS≥2, SOFA≥2 or SOFA_change≥2 [17, 19]. Therefore we introduced additional analyses in our study (Table 4). Our observation that, at infection onset, SOFA was ≥2 in 100% of decedents and also in 99% of survivors, which was similarly reported in the sepsis-3 validation study [9] (98% and 90% respectively), revealed SOFA with the threshold of 2 to yield poor guidance as prognostic criterion in infected ICU patients. High sensitivity for in-hospital mortality of SOFA≥2 at the expense of specificity was reported before [21, 31]. Evaluated by risk ratios and correlation coefficients, SIRS≥2 captured a similar risk for in-hospital mortality compared to SOFA_change≥2 in infected patients. However, while 20% of infected patients that had a change in SOFA <2 points at infection onset died in hospital later on, this was observed in 11% for SIRS <2.

### Infection-specific mortality association patterns of sepsis criteria–A new reference for mortality-based evaluation of sepsis criteria

Further doubt was raised whether mortality was an eligible endpoint for the validation of sepsis criteria [17–21]. To address this, we established references for the correlation between sepsis and in-hospital mortality, which was postulated [26, 27] but omitted during sepsis-3 validation process [9]. Based on sepsis-3 data, the expected correlation could be estimated as 0.10 for infected ICU patients, demonstrating that in-hospital mortality can be used to examine criterion validity of sepsis criteria. But contrary to the postulated way to consider this association, namely the larger the better [2, 9, 37], application of our mathematical probabilistic model prompts that a valid criterion is expected to show a positive but weak correlation with in-hospital mortality in infected patients and no correlation in non-infected patients, as sepsis cannot cause death in non-infected patients.

Consequently, the consideration of non-infected patients in our model revealed that in order to be valid, a sepsis measure must display this infection-specific mortality association capturing two questions: If the criterion is fulfilled (namely SIRS, SOFA and SOFA_change are ≥2, respectively), does it correlate with mortality in infected patients, but not in non-infected patients? And is the displayed correlation with mortality within the mathematical estimation for sepsis in infected patients?

In this study, the expected infection-specific mortality association pattern was solely observed for SIRS≥2, which was—adequate for a sepsis measure—prognostic in infected patients and non-prognostic in non-infected patients (Table 5). This means that for an infected patient the presence of SIRS≥2 significantly worsens the prognosis, and it does so to the extent that is expected for the occurrence of sepsis, while in non-infected patients the presence of SIRS≥2 does not alter the prognosis. Fittingly, risk ratio for in-hospital mortality was significant for SIRS≥2 in infected patients and non-significant in non-infected patients (Table 3). In contrast, SOFA≥2 showed higher correlations with in-hospital mortality in non-infected than in infected patients which contradicted expectations for a valid sepsis criterion. SOFA_change≥2 deviated from expectations for correlation with in-hospital mortality in infected and non-infected patients. In our study, none of the SOFA applications captured the mortality pattern of sepsis.

As was considered before [18, 32, 41], our analysis substantiates, and for the first time establishes by mathematical reasoning, that good mortality prediction and valid sepsis detection are separate issues. This gave reason to investigate an infection-specific mortality association pattern as a validity measure of sepsis criteria. This expected pattern was not displayed by SOFA, SOFA≥2, SOFA_change≥2 or SIRS, but solely by SIRS≥2.

### Limitations and strengths

This was a single center study including 791 patients with higher morbidity compared to the sepsis-3 ICU cohort [9]. Consequently, in-hospital mortality was higher, but in good agreement with results of 11883 patients from 133 German ICUs, in which in-hospital mortality for sepsis was 40% [59] compared to 39% in this study. The higher sepsis mortality in European compared to US or Australian ICUs is topic of ongoing debate [59, 60]. Nevertheless, AUROC analyses for SOFA, SOFA_change and SIRS were in very good agreement with those reported for the sepsis-3 ICU population [9] indicating comparability. We strongly encourage validation of our results in different settings. To our knowledge, we are the first to establish quantifiable references for the correlation of sepsis and mortality. Calculation of reference correlations was based on published sepsis-3 data gained from 7932 ICU patients [9]. By considering non-infected patients in our study, we address a named limitation of sepsis-3 [2, 9].

## Conclusions

In our ICU cohort, application of SOFA≥2 yielded a more liberal sepsis diagnosis than SIRS≥2, diminishing the differentiation between uncomplicated infection and sepsis. In this study, we establish a mathematical probabilistic model as a new approach to mortality-based evaluation of sepsis criteria that substantiates by mathematical reasoning, that good mortality prediction and valid sepsis detection are separate issues. Based on here established references, valid sepsis criteria are characterized by a weak correlation with in-hospital mortality in infected patients and no association in non-infected patients. This expected infection-specific mortality pattern was not displayed by SOFA, SOFA≥2, SOFA_change≥2 or SIRS, but solely by SIRS≥2. Further, our approach revealed that validity evaluation with mortality as endpoint is necessary but not sufficient for validation of sepsis criteria as none of the criteria showed an infection-specific occurrence that would be essential for reliable sepsis detection.

## Supporting information

S1 Appendix (DOCX)

S2 Appendix Spreadsheet on calculations performed in the data of the UPMC ICU validation cohort of sepsis-3 validation study. (XLSX)

S3 Appendix Anonymized data set underlying our study. (XLSX)
