# Supplementary material for: Impact of different consensus definition criteria on sepsis diagnosis in a cohort of critically ill patients—Insights from a new mathematical probabilistic approach to mortality-based validation of sepsis criteria
Source: PLoS One. 2020 Sep 8;15(9):e0238548. doi: 10.1371/journal.pone.0238548 (PMC7478755; doi:10.1371/journal.pone.0238548)
Supplement: S1 Appendix — (DOCX) [file pone.0238548.s001.docx]

**Supporting information**

**S1 Appendix.**

**A Text. Details on definitions**

**B Text. Baseline risk model**

**C Text. Sensitivity analysis**

**D Text. Probabilistic mathematical model of the**

**relation between sepsis and in-hospital**

**death**

**E Text. Supplementary references**

**A. Details on definitions**

**SOFA scoring**

SOFA scoring was performed according to the original SOFA description [1, 2]: The SOFA score ranges from 0 to 24, where 0 to 4 points are assigned for 1 of 6 organ systems: respiratory, hematologic, hepatic, cardiac, neurologic, and renal. The most abnormal measurement in each organ category within a 24 hour period was chosen for scoring. Due to ward organization, not calendar days, but 24 hour periods from 2 pm on one day to 2 pm the prior day were evaluated. In case a patient entered the ICU after 2 pm or was released before 2 pm the fraction of this 24 hour period was considered. For the respiration category EPIC II conversion tables [3] were used in case paO2 or FiO2 were not measured directly. In case of Extra Corporal Membrane Oxygenation (ECMO) therapy 4 points were given in the respiration category. “Respiratory support” was applied as published [4]. Regarding the cardiovascular category, vasopressor use was not counted as cardiovascular dysfunction if the reason for its administration was an artificially high blood pressure aim (in most cases concerning neurosurgical patients). As postulated by Vincent et al. the Glasgow Coma Scale (GCS) score that the patient would have in the absence of sedation [5, 6] was taken into account. In case of renal replacement therapy 4 points were given in that category. For missing measures, last value carried forward was applied.

**SIRS extraction**

SIRS criteria that range from 0 to 4 according to Bone et al. were extracted [7], wherein 1 point is given for perturbations of the following variables combined with the following thresholds: temperature >38°C or <36°C; heart rate >90/min; respiratory rate >20/min or paCO2 <32mmHg (4.3 kPa); White blood cell count >12000/mm^3^ or <4000/mm^3^. The >10% immature bands criterion was not taken into account.

**Identification of infection onset**

For identification of infected encounters we applied the approach chosen in sepsis-3 original publication [8] in order to assure comparability: Exclusively the first episode of (suspected) infection for each encounter was identified as the combination of antibiotics and body fluid cultures. For identification of infection two concurrent events were required: Firstly, order for administration of antibiotics and secondly order for body fluid culture. These two events needed to occur within a specific time frame with two options: Option 1: antibiotic had to be given within 72 hours following first culture. Option 2: Culture had to be drawn within 24 hours following first antibiotic dose. Infection onset was the time point on whichever of these two events occurred first.

We excluded all antibiotic doses administered as a single dose. We included microbiological samples from a variety of sites: abdomen, bronchoalveolar lavage, blood, cerebral spinal fluid, catheters/devices, pleural space, urinary tract. Culture types included bacterial and fungal. Screening swabs were excluded.

**Defining evaluation time points for non-infected patients**

To enable analyses in non-infected patients we sought to define evaluation time points for non-infected patients serving as equivalent to infection onset in infected patients. Therefore, by computational query, we selected the evaluation time points in non-infected patients in such a way that the resulting distribution resembled the distribution of infection onset time points in infected patients as closely as possible. The resulting distribution is displayed in Supplementary Figure A.


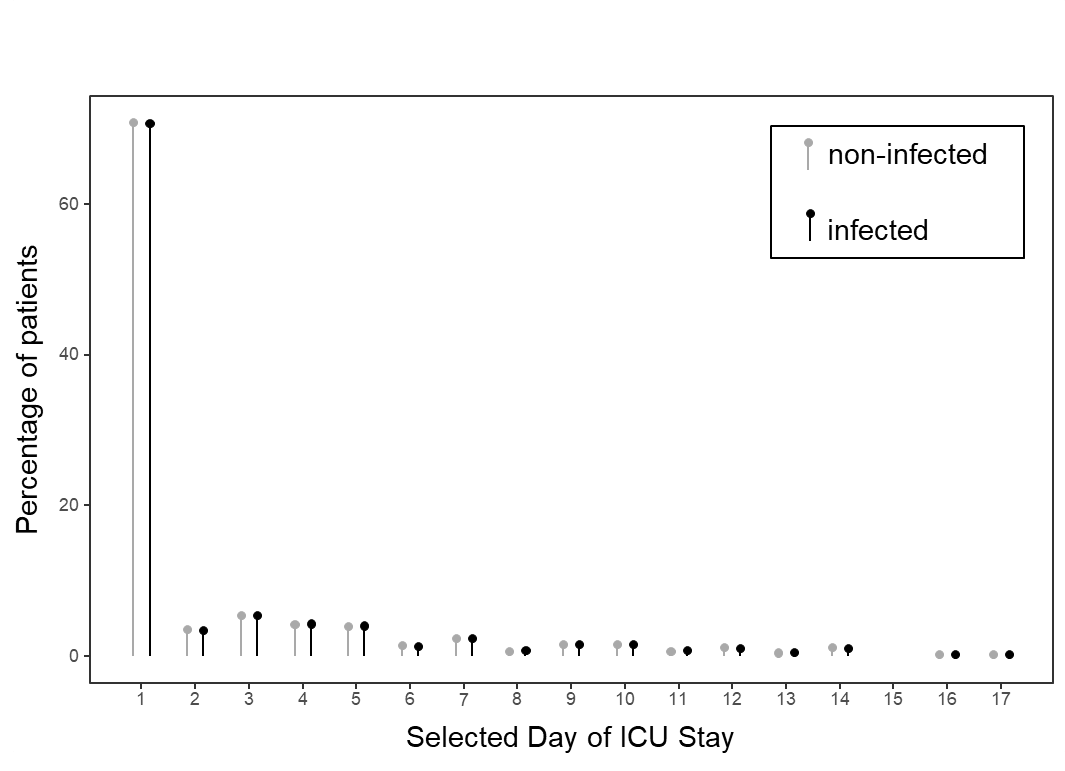
**Fig A. Distribution of time points of infection onset in infected patients and corresponding evaluation time points in non-infected patients**

**SOFA application**

To ensure comparability, we applied the methodology used in the sepsis-3 validation study [8] as closely as possible in our data:

Maximum SOFA: In a first step, two observation periods around infection onset were determined: One period evaluated up to 48 hours before (pre-period) and a second up to 24 hours after (post-period) infection onset. For each of these two periods a SOFA score was determined by choosing the most abnormal measurement within the observed time period. The higher of these two SOFA scores was chosen for maximum SOFA analyses.

Acute change in SOFA: Acute change in SOFA was defined as the difference between post-period and pre-period SOFA [8] with one exception: For patients in whom an infection was identified within 24 hours after ICU admission zero was assumed as baseline SOFA score in case no SOFA score was available in the time period of 48 hours before ICU admission, as recommended [9] and applied in comparable studies [10-12].

For the SOFA operationalizations, one major deviation compared to the sepsis-3 validation study was necessary: In the sepsis-3 validation study, SOFA scores could be calculated at arbitrary time points [8]. In our ICU, we performed SOFA scoring for a patient once a day, as it was originally defined [1]. Due to this circumstance, we had to standardize the time point of ‘infection onset’ to the next SOFA evaluation time point. This slightly changed the considered time windows: Compared to sepsis-3 validation study [8], the pre-period window was shortened and the post-period window was extended due to the time difference between the ‘real’ and ‘standardized’ infection onset. Nevertheless, the real infection onset was certainly not only covered, but in the center of the analyzed time frame.

**B. Baseline risk model**

In the sepsis-3 validation study AUROC analysis was performed for the suggested clinical criterion in combination with a baseline risk model based on pre-infection criteria using fractional polynomial regression [8]. We applied similar baseline risk modeling including age and the weighted Charlson comorbidity score [13-16] (each as a fractional polynomial [17]) as well as sex. Ethnicity was not incorporated due to negligibility in our cohort. We used the R-package ‘mfp’ version 1.5.2 [18]. The application of this procedure resulted in models that contained solely the score in a linear form (SOFA, SOFA_change, SIRS) and no other variable. Consequently, the discriminators obtained from the models are strictly monotone transformations of the scores and thus the Receiver Operating Characteristic Curve is identical with the one obtained from the score.

**C. Sensitivity analysis**

For sensitivity analyses examination was limited to the day of ICU admission (n total=683, n infected=261; n non-infected=422) for two reasons: It entailed fixation for comparison between infected and non-infected patients, and major studies examining sepsis-3 in the ICU limited observation to the 24 hours after admission [10, 11, 19-21].

261 patients (33% of total cohort) entered the ICU with an infection (in-hospital mortality 44%). Results were overall consistent with those reported for the entire cohort. Results are displayed in Table A:

**Table A. Sensitivity analysis: AUROC analysis for particular operationalization and in-hospital mortality limited to the day of ICU admission in comparison to total observation period.**

|  | **Patient Subgroup** | **First day**  **AUROC (95%CI)** | **Total observation period**  **AUROC (95%CI)** |
| --- | --- | --- | --- |
| SOFA | Non-infected | 0.85 (0.80 – 0.90) | 0.85 (0.80 – 0.90) |
|  | Infected | 0.76 (0.70 – 0.82) | 0.75 (0.69 – 0.80) |
| SIRS | Non-infected | 0.61 (0.53 – 0.69) | 0.62 (0.53 – 0.70) |
|  | Infected | 0.62 (0.56 – 0.69) | 0.61 (0.55 – 0.66) |
| SOFA_change | Non-infected | 0.82 (0.77 – 0.88) | 0.74 (0.65 – 0.82) |
|  | Infected | 0.72 (0.65 – 0.78) | 0.70 (0.65 – 0.76) |

95%CI, 95% Confidence Interval; AUROC, Area Under the Receiver Operating Characteristic Curve.

**D. Probabilistic model of the relation between sepsis and in-hospital Death**

In the following we provide a formalistic assessment of the probabilistic relation between sepsis and in-hospital death. The resulting benefit is a formula describing the correlation between both variables. Along with easily obtainable empirical data, this formula allows to estimate the extent of the correlation between sepsis and in-hospital death.

The importance to set expectations about the size of this agreement, when conducting criterion validity experiments was highlighted in the ‘framework for the development and interpretation of different sepsis definitions and clinical criteria’ [22, 23]. We demonstrate in the following that an estimate of this relation is a necessary reference if mortality is used to investigate the criterion validity of a proposed sepsis measure. In order to display validity of the proposed sepsis measure, its association with in-hospital death should be of approximately the same size as the estimated reference correlation of sepsis and in-hospital death.

In a further application, this model allows evaluation of the rational to use the relation between the proposed sepsis measure and in-hospital death during the validation process of sepsis-3 along with the applied judgment, namely that larger associations indicate greater validity of a sepsis measure [8, 9]. Following this approach, the question whether a strong association between sepsis and in-hospital death is sufficient to characterize a sepsis measure, is assessed.

**Formalistic model and notation**

For the purpose of our analysis we regard cause unspecific in-hospital death and sepsis (the event of having sepsis at least once during a hospitalization) as dichotomous random variables. Let $\text{P}\left( deceased \right)$ denote the probability of dying during hospitalization and $\text{P}\left( septic \right)$ denote the probability that the patient has sepsis at least once during his hospitalization — in short $\text{P}\left( deceased \right)$ is the in-hospital mortality and $\text{P}\left( septic \right)$is the in-hospital sepsis prevalence. The bivariate distribution of in-hospital death and sepsis can be expressed in a fourfold table, where $\text{P}\left( X \right)$ denotes the probability of event$X$ (Supplementary Figure B).

|  |  | **in-hospital death** | |  |
| --- | --- | --- | --- | --- |
|  |  | deceased | survived | sum |
| **sepsis** | septic | $\text{P}\left( deceased and septic \right)$ | $\text{P}\left( survived and septic \right)$ | $\text{P}\left( septic \right)$ |
|  |  |  |  |  |
|  | non-septic | $\text{P}\left( deceased and non-septic \right)$ | $\text{P}\left( survived and non-septic \right)$ | $\text{P}\left( non-septic \right)$ |
|  |  |  |  |  |
|  | sum | $\text{P}\left( deceased \right)$ | $\text{P}\left( survived \right)$ | $1$ |

**Fig B. Bivariate distribution of in-hospital death and sepsis.**

The most commonly used measure in validation research that expresses the association between measures or a measure and a criterion is Pearson’s product moment correlation coefficient. In terms of the probabilities of the bivariate distribution the theoretical correlation between in-hospital mortality and sepsis $\text{ρ}\left( death, sepsis \right)$ is

| $\text{ρ}\left( death, sepsis \right)=\frac{\text{P}\left( deceased and septic \right)- \text{P}\left( deceased \right)\text{P}\left( septic \right)}{\sqrt{\text{P}\left( deceased \right)\left( 1-\text{P}\left( deceased \right) \right)\text{P}\left( septic \right)\left( 1-\text{P}\left( septic \right) \right)}}$. | (1) |
| --- | --- |

This equation indicates that for any values of hospital mortality and sepsis prevalence, the size of the correlation is determined by $\text{P}\left( deceased and septic \right)$, the probability to die in hospital and being diagnosed (at least once) with sepsis. Larger values of this probability lead to larger correlations between in-hospital death and in-hospital sepsis.

**Application of the model to derive reference values for infected ICU patients**

As it constitutes the currently recommended definition and operationalization of sepsis by the Society of Critical Care Medicine (SCCM) and the European Society of Intensive Care Medicine (ESICM) [9], we chose sepsis-3 to determine the sepsis status of a patient, and used numbers from the sepsis-3 validation study [8] to calculate the bivariate distribution (Fig C). Filling these numbers into equation 1 resulted in an estimated correlation between sepsis and in-hospital death of 0.104 for the population of infected ICU patients. This quantified reference served as reference for statistical examination of the observed correlations in our ICU cohort.

|  |  | **in-hospital death^a^** | |  |
| --- | --- | --- | --- | --- |
|  |  | deceased | survived | sum |
| **Sepsis^a^** | septic | $\frac{1263}{7932}\approx0.1592$ | $\frac{5979}{7932}\approx0.7538$ | $\frac{7242}{7932}\approx0.9130$ |
|  |  |  |  |  |
|  | non-septic | $\frac{26}{7932}\approx0.0033$ | $\frac{664}{7932}\approx0.0837$ | $\frac{690}{7932}\approx0.0870$ |
|  |  |  |  |  |
|  | sum | $\frac{1289}{7932}\approx0.1625$ | $\frac{6643}{7932}\approx0.8375$ | $\frac{7932}{7932}=1$ |

**Fig C. Distribution of in-hospital death and sepsis for the primary sepsis-3 Intensive Care Unit (ICU) validation cohort.**

^a^based on numbers published in sepsis-3 validation study for the University of Pittsburg Medical Center (UPMC) ICU validation cohort (see eTable 3 in supplement of [8]; SOFA≥2 was taken as septic condition; for further details on calculations performed please find the spreadsheet in S2 Appendix)

**Application of the model to derive reference values for non-infected ICU patients**

As the presence of an infection is a necessary condition for sepsis, non-infected patients cannot suffer from sepsis. Thus for non-infected patients $\text{P}\left( septic \right)=0$, and one would reasonably state that sepsis and death cannot be correlated, meaning that the correlation coefficient between death and sepsis equals zero in the subpopulation of non-infected patients.

The latter statement can also be demonstrated via equation 1. The fact that $0\leq\text{P}\left( deceased and septic \right)\leq\text{P}\left( septic \right)$ allows to bracket $\text{ρ}\left( death, sepsis \right)$. The resulting upper and lower bound equal 0 when $\text{P}\left( septic \right)=0$ and thus $\text{ρ}\left( death, sepsis \right)=0$ in this case.

**Formal assessment of the fundamental assumption made by the sepsis-3 task force to justify the usage of in-hospital death as endpoint for criterion validity evaluation of sepsis criteria**

In the following, considerations are limited to infected patients as performed in the sepsis-3 validation study [8]. Let us assume that the sepsis prevalence and the mortality of sepsis is less than one and bigger than zero for infected patients. Informally stated, this means that not every infected patient is septic (but some are) and that not every septic patient dies in hospital.

The task force justified the usage of in-hospital death as endpoint for criterion validity evaluation by the assumption that death is far more common in infected patients who have sepsis than in those who do not. This assumption can be formally expressed by conditional probabilities as

| $\text{P}\left( deceased \vert septic \right)\gg\text{P}\left( deceased \vert non- septic \right).$ | (2) |
| --- | --- |

As shown in equation 1 the crucial quantity is $\text{P}\left( deceased and septic \right)$, the probability to die in hospital and being diagnosed (at least once) with sepsis. We can connect equation 2 to the relevant probabilities of the bivariate distribution in the following way

| $\text{P}\left( deceased and septic \right)=\text{P}\left( Death \vert septic \right)\text{ P}\left( septic \right)$ | (3) |
| --- | --- |

and

| $\text{P}\left( deceased and non-septic \right)=\text{P}\left( Death \vert non-septic \right)\left( 1-\text{P}\left( septic \right) \right)$. | ( 4) |
| --- | --- |

Equations 3 and 4 reveal that the assumption underlying the sepsis-3 validation process does not allow us to draw any conclusion about the correlation between in-hospital death and sepsis, because the true value of $\text{P}\left( septic \right)$ remains unknown. Thus, the relative statement on the conditional probabilities formulated in sepsis-3 validation process (equation 2) is not sufficient to draw conclusions about the validity of a sepsis measure.

A patient who was infected and died later was probably septic, but by far not every septic patient dies. Thus, the expected correlation of sepsis and in-hospital death cannot be very large. Therefore, the here established reference correlation is of crucial importance for evaluating the validity of sepsis criteria if using in-hospital mortality as endpoint.

**E. Supplementary References**

1. Vincent J-L, Moreno R, Takala J, Willatts S, De Mendonça A, Bruining H, et al. The SOFA

(Sepsis-related Organ Failure Assessment) score to describe organ dysfunction/failure. Intensive Care Med. 1996;22: 707-710.

1. Vincent J-L, De Mendonça A, Cantraine F, Moreno R, Takala J, Suter PM, et al. Use of the SOFA score to assess the incidence of organ dysfunction/failure in intensive care units: results of a multicenter, prospective study. Crit Care Med. 1998;26: 1793-1800.
2. Vincent J-L, Rello J, Marshall J, Silva E, Anzueto A, Martin CD, et al. International study of

the prevalence and outcomes of infection in intensive care units. JAMA. 2009;302: 2323-2329.

1. Tallgren M, Bäcklund M, Hynninen M. Accuracy of Sequential Organ Failure Assessment

(SOFA) scoring in clinical practice. Acta Anaesthesiol Scand. 2009;53: 39-45.

1. Vincent J-L, Takala J, Moreno RP, Sakr Y, Marshall JC. The Richmond Agitation-Sedation

Scale Should Not Be Used to Evaluate Neurologic Function. Crit Care Med. 2016;44: e450.

1. Ferreira FL, Bota DP, Bross A, Mélot C, Vincent J-L. Serial evaluation of the SOFA score to predict outcome in critically ill patients. JAMA. 2001;286: 1754-1758.
2. Bone RC, Balk RA, Cerra FB, Dellinger RP, Fein AM, Knaus WA, et al. Definitions for sepsis and organ failure and guidelines for the use of innovative therapies in sepsis. The ACCP/SCCM Consensus Conference Committee. American College of Chest Physicians/Society of Critical Care Medicine. Chest. 1992;101: 1644-1655.
3. Seymour CW, Liu VX, Iwashyna TJ, Brunkhorst FM, Rea TD, Scherag A, et al. Assessment of clinical criteria for sepsis: for the Third International Consensus Definitions for Sepsis and Septic Shock (Sepsis-3). JAMA. 2016;315: 762-774.
4. Singer M, Deutschman CS, Seymour CW, Shankar-Hari M, Annane D, Bauer M, et al. The

Third International Consensus definitions for sepsis and septic shock (Sepsis-3). JAMA. 2016;315: 801-810.

1. Raith EP, Udy AA, Bailey M, McGloughlin S, MacIsaac C, Bellomo R, et al. Prognostic accuracy of the SOFA score, SIRS criteria, and qSOFA score for in-hospital mortality among adults with suspected infection admitted to the intensive care unit. JAMA. 2017;317: 290-300.
2. Shankar-Hari M, Harrison D, Rubenfeld G, Rowan K. Epidemiology of sepsis and septic

shock in critical care units: comparison between sepsis-2 and sepsis-3 populations using a national critical care database. Br J Anaesth. 2017;119: 626-636.

1. Fullerton JN, Thompson K, Shetty A, Iredell JR, Lander H, Myburgh JA, et al. New sepsis

definition changes incidence of sepsis in the intensive care unit. Crit Care Resusc. 2017;19: 9-13.

1. Quan H, Sundararajan V, Halfon P, Fong A, Burnand B, Luthi J-C, et al. Coding algorithms for defining comorbidities in ICD-9-CM and ICD-10 administrative data. Med Care. 2005;43: 1130-1139.
2. Charlson ME, Pompei P, Ales KL, MacKenzie CR. A new method of classifying prognostic

comorbidity in longitudinal studies: development and validation. J Chronic Dis. 1987;40: 373-383.

1. Halfon P, Eggli Y, van Melle G, Chevalier J, Wasserfallen J-B, Burnand B. Measuring potentially avoidable hospital readmissions. J Clin Epidemiol. 2002;55: 573-587.
2. Sundararajan V, Quan H, Halfon P, Fushimi K, Luthi J-C, Burnand B, et al. Cross-national comparative performance of three versions of the ICD-10 Charlson index. Med Care. 2007;45: 1210-1215.
3. Royston P, Sauerbrei W. Building multivariable regression models with continuous covariates in clinical epidemiology with an emphasis on fractional polynomials. Methods Inf Med. 2005;44: 561-571.
4. Ambler G, Benner A. Mfp: Multivariable Fractional Polynomials. R package version 1.5.2.

2015; Available from: https://CRAN.R-project.org/package=mfp.

1. Costa RT, Nassar Jr AP, Caruso P. Accuracy of SOFA, qSOFA, and SIRS scores for mortality in cancer patients admitted to an intensive care unit with suspected infection. J Crit Care. 2018;45: 52-57.
2. Fang X, Wang Z, Yang J, Cai H, Yao Z, Li K, et al. Clinical Evaluation of Sepsis-1 and Sepsis-3 in the ICU. Chest. 2018;153: 1169-1176.
3. Cheng B, Li Z, Wang J, Xie G, Liu X, Xu Z, et al. Comparison of the performance between

sepsis-1 and sepsis-3 in ICUs in China: a retrospective multicenter study. Shock. 2017;48: 301-306.

1. Angus DC, Seymour CW, Coopersmith CM, Deutschman CS, Klompas M, Levy MM, et al. A framework for the development and interpretation of different sepsis definitions and clinical criteria. Crit Care Med. 2016;44: e113-e121.
2. Seymour CW, Coopersmith CM, Deutschman CS, Gesten F, Klompas M, Levy M, et al. Application of a framework to assess the usefulness of alternative sepsis criteria. Crit Care Med. 2016;44: e122-e130.
